# How Do Gait Outcomes Evolve in Adults with Spastic Cerebral Palsy Who Received Orthopedic Treatment in Childhood?

**DOI:** 10.3390/children13010158

**Published:** 2026-01-22

**Authors:** Anne Tabard-Fougère, Alice Bonnefoy-Mazure, Geraldo de Coulon, Oscar Vazquez, Stéphane Armand

**Affiliations:** 1 Pediatric Orthopedic and Traumatology Unit, Geneva University Hospitals, University of Geneva, 1205 Geneva, Switzerland; 2 Kinesiology Laboratory, Geneva University Hospitals, University of Geneva, 1205 Geneva, Switzerland; 3 Centre of Research on Skeletal Muscle and Movement, Geneva University Hospitals, University of Geneva, 1205 Geneva, Switzerland

**Keywords:** cerebral palsy, gait analysis, motor decline, adult outcomes, longitudinal study, walking speed, kinematics

## Abstract

**Highlights:**

**What are the main findings?**

• Individual trajectories in adulthood diverge by cerebral palsy (CP) type, with those with bilateral CP GMFCS II and III showing a higher frequency of functional decline from early adulthood to adulthood life stages.
• Normalized walking speed declines significantly for all individuals with CP.

**What are the implications of the main findings?**

• The findings challenge the assumption that gait improvements in childhood are sustained, revealing more frequent functional decline in adulthood, especially for individuals with bCP GMFCS II and III.
• This study underscores the necessity for lifelong, regular monitoring and potential intervention for adults with CP, particularly those with greater motor impairments.

**Abstract:**

**Background/Objectives**: Cerebral palsy (CP) is the most common cause of physical disability in childhood. While gait improvements are often observed during childhood, it remains unclear whether these gains are sustained into adulthood. This study aimed to evaluate the long-term evolution of gait outcomes from childhood to adulthood in individuals with CP who received orthopedic care early in life. **Methods**: This retrospective study included 83 adults with cerebral palsy (44 unilateral/uCP, 39 bilateral/bCP; GMFCS I–III) who underwent clinical gait analysis in childhood and again as adults (minimum 4 years between visits, n = 249 CGA). Gait was assessed using the modified Gait Profile Score (mGPS) and normalized walking speed (NWS). The effects of life stage (childhood, adolescence, early adulthood, and adulthood) were analyzed using Kruskal–Wallis tests with post hoc comparisons. Individual clinical transitions were quantified from early adulthood to adulthood, with a minimal clinically important difference (MCID) change in mGPS (1.6°) and NWS (0.20 s^−1^) for improvement or decline. **Results**: Longitudinal analysis revealed that while group-average mGPS improved from childhood to adulthood, NWS declined significantly for all patients (*p* < 0.01). However, individual trajectories from early adulthood to adulthood diverged by CP type. Those with bCP GMFCS II and III had a more frequent clinical decline in mGPS (4/14, 29%), with minimal potential for improvement (1/14, 17%). In contrast, individuals with uCP had less frequent decline (1/17, 6%) and a greater improvement (3/17, 18%). **Conclusions**: While significant improvements in gait quality are achieved by early adulthood, substantial clinical decline occurs during adulthood in bCP (GMFCS II–III) patients. These findings highlight the need for lifelong monitoring, with re-evaluation regarding the need for surgical interventions from early adulthood to adulthood in bCP patients with greater motor impairments.

## 1. Introduction

Cerebral palsy (CP) is the most common cause of physical disability in childhood, with a global birth prevalence ranging from 1.6 to 3.4 per 1000 births [1]. CP is characterized by permanent motor disorders resulting from non-progressive disturbances in the developing brain [2,3]. Among the motor impairments, gait dysfunction is a major contributor to long-term morbidity [4,5]. Without intervention, these motor impairments naturally progress, leading to a decline in walking ability and quality of life [6].

Due to the heterogeneity of CP disorders, treatment must be personalized. There are a variety of possible interventions (e.g., physiotherapy, orthoses, botulinum toxin injection, rhizotomy, single-level surgery (SLS), and single-event multilevel surgery (SEMLS)), which can sometimes be combined. Orthopedic interventions are central to the management of gait abnormalities in CP, aiming to optimize function while preventing or minimizing gait deviations and secondary musculoskeletal complications [2,3]. Several longitudinal studies have demonstrated that early orthopedic treatment can result in significant and durable improvements in gait and functional mobility, with gains maintained into adolescence and early adulthood [3,7].

Long-term outcomes beyond early adulthood are incompletely understood, as highlighted by multiple reviews and cohort studies. A systematic review identified that at least 25% of ambulant adults with CP experience early mobility decline, with a higher risk linked to bilateral motor impairment, older age, and greater pain or fatigue [8]. However, the generalizability of these findings is limited by methodological issues, including a predominance of young participants (mean age < 40 years in 13 of 16 studies), infrequent reporting of time intervals (10/16 studies), and reliance on patient-reported walking ability data. More recent cohort studies of adults aged 25–45 years who received pediatric orthopedic care report that most maintain functional gains and stable gait kinematics into their third decade, with only a minority developing new or worsening deformities that impact function [9,10,11,12]. The risk of residual deformity and functional decline is influenced by factors such as CP subtype, Gross Motor Function Classification System (GMFCS) level, and the timing of intervention [2,3].

Gait decline in adults with CP could arise from primary neuromuscular impairments (muscle weakness, increased passive stiffness, or impaired selective motor control), reduced energy efficiency, or musculoskeletal aging with early-onset osteoarthritis and osteoporosis [2,12]. Clinically, gait deterioration significantly impacts participation and quality of life, with mobility decline associated with reduced physical function, diminished social participation, increased falls, pain, and fatigue [2,12]. It is important to distinguish between maintenance of gait pattern (preserving qualitative aspects such as step length, cadence, and joint kinematics) and maintenance of walking capacity (the ability to perform walking tasks over distance or time), as these represent distinct aspects of mobility that may decline independently [12].

The literature consistently highlights a lack of systematic, large-scale longitudinal studies tracking motor function and mobility into later adulthood and calls for further research to address these gaps [3].

While these studies provide a crucial foundation, key questions remain regarding the objective trajectory of gait quality into mid-adulthood and the differential impact of CP subtype. Specifically, longitudinal studies with instrumented gait analysis [9,10,11,12] have demonstrated stability in early adulthood but have not extensively tracked kinematic outcomes beyond the fourth decade or directly compared the long-term trajectories between unilateral (uCP) and bilateral (bCP) subtypes using objective biomechanical measures.

Thus, the present study aims to address this gap by evaluating the trajectory of gait outcomes from childhood to adulthood in patients with unilateral (uCP) and bilateral (bCP) cerebral palsy who received orthopedic treatments (including physiotherapy, orthosis, botulinum toxin injection, surgical treatments, etc.) during childhood. We hypothesized that CP subtype is a critical determinant of long-term gait quality, with individuals with uCP expected to maintain stable gait kinematics across life stages, while those with bCP expected to demonstrate a significantly higher proportion of clinically meaningful gait deterioration over time.

## 2. Materials and Methods

### 2.1. Study Design and Population

This retrospective longitudinal cohort study included patients with CP who underwent clinical gait analysis (CGA) in the Kinesiology Laboratory of a tertiary hospital between 1994 and 2025. The study was approved by the local ethics committee (CER no. 2018–00229). Gross motor function for all participants was classified according to the Gross Motor Function Classification System (GMFCS). To ensure consistent and accurate classification across the entire study period (1994–2025), GMFCS levels were determined through a structured retrospective video review. Based on the diagnosis, the sample was divided into two groups: unilateral CP (uCP) and bilateral CP (bCP).

The inclusion criteria were (1) diagnosis of spastic CP with GMFCS level I to III; (2) an initial CGA in childhood (<18 years of age); (3) a follow-up CGA in adulthood (≥18 years of age); and (4) a minimum interval of 4 years between the first and last CGA.

The exclusion criteria included orthopedic surgery or neurological intervention within 12 months preceding any gait analysis, botulinum toxin injection (BTX) 6 months prior to the CGA, or the presence of other than spastic CP neurological/musculoskeletal conditions confounding gait.

Life stages were defined as childhood (female: <13.5 years; male: <15.5 years), adolescence (female: 13.5–18 years; male: 15.5–18 years), early adulthood (18–25 years), and adulthood (>25 years). For each patient, CGA were compared across these stages [13,14].

### 2.2. Gait Evaluation

Participants were instructed to walk barefoot at a comfortable, self-selected speed along a 10 m walkway. Kinematic parameters were measured using a 12-camera motion analysis system (model Oqus 7+, Qualisys, Göteborg, Sweden) between 2015 and 2019, a 12-camera motion analysis system (Vicon MX3+, Vicon Peak, Oxford, UK) between 2007 and 2015, and a 6-camera motion analysis system (Vicon 460, Vicon Peak, Oxford, UK) before 2007. The marker trajectories were recorded at 100 Hz and filtered using the predicted mean-squared error filter MSE10 in the Nexus software 1.8.2.49787h before 2015 and high-pass 4th order Butterworth filter (10 Hz) after. Participants were equipped with 35 reflective markers that were placed on the skin at defined anatomical and technical landmarks according to the full-body Conventional Gait Model [15]. To ensure consistency for longitudinal comparison, the raw three-dimensional marker trajectories from all sessions were reprocessed using a single, standardized software (Moveck^® ^ 0.7.2, https://moveck.com) that replicates the Conventional Gait Model.

### 2.3. Orthopedic Treatments

Based on patient-specific indications and good clinical practice at the time of inclusion, all participants received orthopedic treatments for CP during childhood and adolescence, which were multidisciplinary and individualized, integrating surgical and non-surgical interventions to address musculoskeletal impairments and support functional outcomes [3]. Data on orthopedic surgical history were collected retrospectively for all participants from the institutional surgical registry and clinical notes. There are a variety of possible interventions (e.g., physiotherapy, orthoses, botulinum toxin injection, SLS, and SEMLS), which can sometimes be combined. The total number of procedures per participant was calculated and used as the number of surgical events.

The specific combination, sequence, and timing of these interventions varied across the cohort. While this heterogeneity is an inherent characteristic of longitudinal studies reflecting real-world clinical practice, it is an important consideration for interpreting the study’s findings, which involves evaluating the overall trajectory following a complete course of pediatric orthopedic care rather than the effect of a single, isolated procedure.

### 2.4. Primary Outcomes

The primary outcomes focused on the evolution of gait quality and function across life stages (childhood, adolescence, early adulthood, and adulthood). For the gait parameters, the affected leg was kept for analysis in uCP, and the mean value of the legs was kept for analysis in bCP.

• **Gait quality:** Assessed using the modified Gait Profile Score (mGPS). This composite kinematic score is computed from eight kinematic lower-limb variables (pelvic tilt, obliquity, rotation, hip flexion, abduction, knee flexion, ankle flexion, and foot angle progression) and represents the root mean square distance between the patient’s kinematic curve and their mean normative curve, expressed in degrees [16].
• **Gait function**: Assessed using normalized walking speed (NWS) in s^−1^, calculated as the ratio of walking speed (m·s^−1^) to leg length (m), to control for the confounding effects of growth on self-selected walking speed [17]. NWS is reported to facilitate direct comparison across individuals of different statures and ages [18].

For a deeper analysis of gait quality evolution, a minimal clinically important difference (MCID) of 1.6° for the mGPS [19] and 0.20 for the NWS [20] was applied. Using these thresholds, patients were categorized for each measure between early adulthood and adulthood. For the mGPS, a lower score indicates improvement, while for the NWS, a higher score indicates improvement. Accordingly, patients were classified as having meaningful improvement (change ≥ MCID in the improving direction), meaningful decline (change ≥ MCID in the declining direction), or stability (change < |MCID|).

### 2.5. Statistical Analysis

All statistical analyses were performed using R software (version 4.5.0, R Foundation for Statistical Computing, Vienna, Austria). Statistical significance was set at *p* < 0.05.

Demographics and clinical characteristics at the first visit were compared using unpaired Student *t*-tests between the uCP and bCP groups, reported with 95% confidence intervals (95%CI) and effect size (ES: r or Cohen’s d); data were reported as mean (standard deviation: SD). The cumulative count of surgical events at the last visit was compared between the uCP and bCP groups.

The effects of life stage (childhood, adolescence, early adulthood, and adulthood) on gait parameters were analyzed separately for the uCP and bCP groups. In these longitudinal analyses, a participant contributed data only for the specific stages in which they were assessed; missing a stage resulted in exclusion from that specific stage’s comparison but not from others. This per-stage, available-case approach was used without imputation. We acknowledge this as a potential limitation, as it could introduce bias if data were not missing at random with respect to clinical outcomes. This led to different sample sizes for each life stage, with a particular reduction in included patients for adulthood (n = 17 uCP, n = 14 bCP). Consequently, the Kruskal–Wallis tests were used to assess global effects across life stages, followed by post hoc paired Wilcoxon tests for pairwise comparisons, with data reported as median [interquartile range: IQR]. To evaluate attrition bias, a sensitivity analysis was performed by comparing baseline (childhood) demographic, clinical, and gait characteristics between participants with data in the adulthood stage (n = 33) and those with data only in the early adulthood stage (n = 50) using the Mann–Whitney U and chi-square tests.

Finally, the chi-square test was used to compare the proportion of participants with clinically meaningful worsening between groups from early adulthood to adulthood.

## 3. Results

### 3.1. Population Characteristics

**Figure 1:**
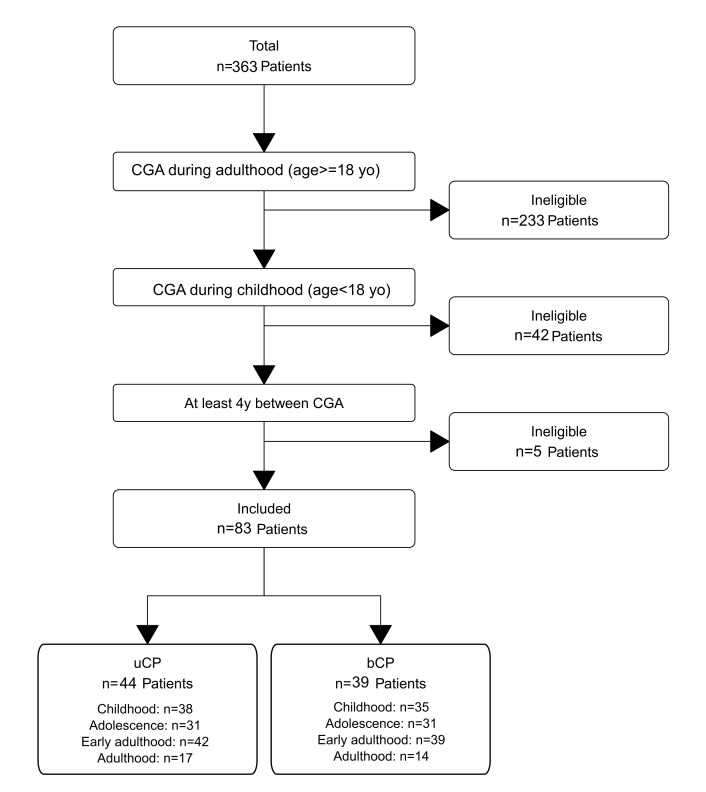
Patient selection flowchart.

From an initial cohort of 363 patients, 83 adults with cerebral palsy (44 unilateral/uCP, 39 bilateral/bCP) met the inclusion criteria of having clinical gait analysis (CGA) both during childhood and adulthood, with a minimum interval of four years between assessments (Figure 1). For the longitudinal analysis across four life stages, the number of available CGA sessions per stage varied, with sample sizes as follows: childhood (n = 38 uCP, 35 bCP), adolescence (n = 31 uCP, 31 bCP), early adulthood (n = 42 uCP, 39 bCP), and adulthood (n = 17 uCP, 14 bCP).

The participants’ characteristics at the first and the last visit were reported in Table 1. The mean time between the first and the last CGA was 13.8 (5.3) years in uCP and 13.9 (6.0) years in bCP, and the mean number of included visits was 3.0 (0.6) (maximum one per life stage). At baseline, the bCP group demonstrated a greater proportion of patients with GMFCS II–III (17/39, 44%), while all patients in the uCP group had GMFCS level I. Within the bCP group, 7 of 39 patients (18%) experienced a change in their GMFCS level between childhood and adulthood. The most common change was a decline in function (6/7 (86%)), with four patients moving from level I to II and one from level II to III. Notably, one patient improved from level II to I, and another transitioned from level II to III later in life, between early adulthood and adulthood.

Throughout the follow-up period, the bCP group required significantly more surgical interventions, with a greater proportion undergoing more than one surgery (51% vs. 23%, *p* = 0.013). As detailed in Table 1, the surgical events varied by CP subtype, with a significantly higher rate of patellar-lowering procedures observed in the bilateral CP group compared to the unilateral CP group (26% vs. 2%, *p* = 0.005). Analysis of gait outcomes showed that while NWS deteriorated in a similar majority in both groups (64% bCP vs. 59% uCP, *p* = 0.809), gait quality as measured by the mGPS was significantly worse in the bCP group compared to the uCP group (*p* = 0.003) at baseline. A greater proportion of individuals in the bCP group experienced a clinically meaningful deterioration in gait quality (13% vs. 5%), although this difference was not statistically significant (*p* = 0.338).

As reported in Table 2, the uCP and bCP groups were well-matched in age at each life stage. A sensitivity analysis was also conducted to assess potential bias due to the reduced sample size in adulthood. Baseline characteristics were compared between participants with data in adulthood (n = 33, 14 uCP and 19 bCP, GMFCS: I: n = 28, II: n = 4, III: n = 1) and those without data in adulthood (n = 50, 25 uCP and 25 bCP GMFCS: I: n = 38, II: n = 6, III: n = 6). The CP type and the GMFCS level distribution were comparable (*p* > 0.05). Critically, no significant differences were found in childhood gait quality (mGPS: median 9.25° vs. 8.68°, *p* = 0.734) or gait function (NWS: median 1.40 s^−1^ vs. 1.58 s^−1^, *p* = 0.765).

### 3.2. Gait Quality Evolution (mGPS)

As reported in Table 1, throughout the follow-up period, the mGPS significantly decreased in both groups (uCP: 8.7 (2.4)° to 7.1 (1.3)°, mean difference= 1.6° [95%CI: 0.9 to 2.3]; *p* < 0.001; bCP: 11.3 (4.8)° to 9.8 (4.4)°, mean difference= 1.5° [95%CI: 0.4 to 2.7], *p* = 0.011). The mGPS declined in a small proportion of patients in both groups (uCP: 2/44 (5%) decline (≥1.6° increase); bCP: 5/39 (13%) decline (≥1.6° increase, *p* = 0.338). The uCP group demonstrated consistently better gait quality (lower mGPS) compared to the bCP group across all life stages (Table 2).

**Figure 2:**
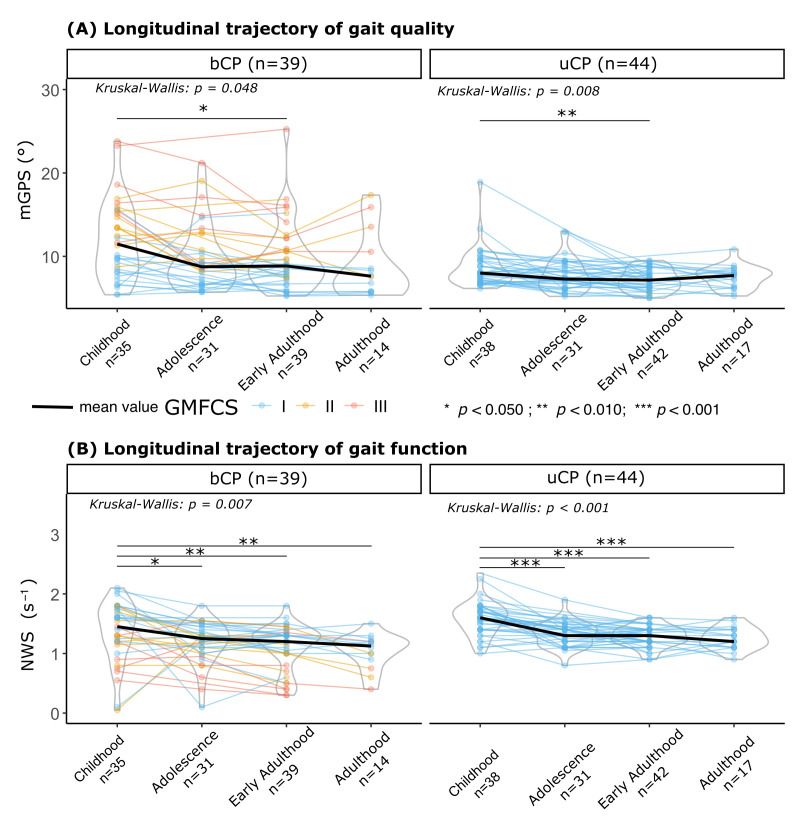
Longitudinal trajectories of gait at different life stages in patients with unilateral (uCP) and bilateral (bCP). (**A**) Gait quality measured using the modified Gait Profile Score (mGPS in °); (**B**) gait function measured using normalized walking speed (NWS in s^−1^), defined as the ratio between the self-selected walking speed (m·s^−1^) and leg length (in m). Statistical significance of the change across life stages within each group was assessed using the Kruskal–Wallis test (* *p* < 0.05, ** *p* < 0.01, *** *p* < 0.001). In these longitudinal analyses, a participant contributed data only for the specific stages in which they were assessed; missing a stage resulted in exclusion from that specific stage’s comparison but not from others. Sample sizes for each life stage are annotated in x-axis labels. GMFCS levels are indicated by the colors.

More specifically, the longitudinal analysis using the Kruskal–Wallis test confirmed a significant effect of life stage in both groups (uCP: *p* = 0.008, bCP = 0.048). The post hoc comparison revealed that mGPS improved only from childhood to early adulthood in both groups (Figure 2A). The change from childhood to adulthood was notable but marginally non-significant (uCP: *p* = 0.079, bCP: *p* = 0.055). Regarding individual trajectories from early adulthood to adulthood, for both groups, there were no significant changes in mGPS. However, based on a MCID of 1.6°, results diverged by CP type. Most of the uCP patients had stable mGPS values, with only one patient showing an MCID decline (1/17, 6%) and three showing an improvement (3/17, 18%). In contrast, bCP patients had a more frequent MCID decline in mGPS (4/14, 29%), with minimal potential for improvement (1/14, 17%). It was notable that all the bCP patients with MCID decline had GMFCS level II or III.

### 3.3. Gait Function Evolution (Normalized Walking Speed)

As reported in Figure 2B, a significant progressive decline in normalized walking speed was observed across life stages for both groups (Kruskal–Wallis: bCP, *p* < 0.001; uCP, *p* = 0.007). The bCP group walked significantly slower than the uCP group at all stages.

Throughout the follow-up period, the walking speed significantly decreased in the uCP (childhood: 1.54 (0.30) s^−1^; adulthood: 1.28 (0.17) s^−1^, mean difference = 0.26 s^−1^ [95%CI: 0.17 to 0.35]; *p* < 0.001; 23/44 (52%) deteriorated (≥0.20 s^−1^ decrease)) and in the bCP (childhood: 1.34 (0.49) s^−1^; adulthood: 1.05 (0.38) s^−1^, mean difference = 0.29 s^−1^; [95%CI: 0.12 to 0.46]; *p* = 0.001; 24/39 (62%) deteriorated (≥0.20 s^−1^ decrease)) groups. However, individual trajectories from early adulthood to adulthood, based on an MCID of 0.20 s^−1^ for NWS, remained rare (bCP: 0/14 (0%), uCP: 3/17 (18%)).

## 4. Discussion

This retrospective long-term study provides a unique longitudinal perspective on gait trajectory in CP from childhood to adulthood at different life stages and stratified by subtypes. Our findings confirm that the CP subtype is a critical determinant of lifelong mobility. The hypothesis was validated; the study revealed a divergent trajectory for gait quality, with the uCP group maintaining stable kinematics and the bCP group showing a higher frequency of functional deterioration. In contrast, gait function (normalized walking speed) exhibited a significant and progressive decline across life stages in both groups, indicating a universal and pervasive challenge to functional mobility.

The preservation of gait kinematics in the uCP group aligns with the optimistic narrative that early, well-managed orthopedic care can yield durable results for a significant portion of this population [9]. These patients, all GMFCS I, likely achieved a musculoskeletal alignment that was mechanically sustainable into adulthood. In contrast, the higher likelihood of deterioration in the bCP group (29%) underscores the more complex and progressive nature of gait pathology in bilateral involvement. This finding is consistent with studies reporting higher mechanical work and energy cost during gait in bCP, which may predispose the individual to earlier decline [8,21]. This decline, which was more pronounced in those with higher GMFCS levels, is consistent with existing literature reporting reductions in walking speed, stride length, and passive range of motion, particularly knee extension and ankle dorsiflexion [10,22]. Supporting this trend within our bCP cohort, 7 of 39 (18%) patients experienced a change in GMFCS level from childhood to adulthood. Most changes represent a functional decline (86%). Four patients moved from level I to II and two from level II to III. Notably, one declined later, between early adulthood and adulthood. A single patient improved from level II to I. The greater surgical burden in the bCP group (51% vs. 23%) further reflects this complexity and suggests that even repeated surgical interventions may not fully halt the progression of gait quality.

Concerning normalized walking speed, the present results showed a progressive, age-related decline in both the uCP and bCP groups, while the bCP patients walked significantly slower at all life stages. In comparison, in typically developing children, normalized walking speed decreases from early childhood [13,23,24], plateaus in late childhood/adolescence, and remains stable through adulthood. The typical age at which both gait speed and normalized gait speed begin to decline in healthy adults is 60–65 years [14,25]. In contrast, adults with CP, even those at GMFCS I–II, walk significantly slower than their healthy peers and show earlier and continuous declines in walking speed [10,12]. This accelerated decline in walking speed in CP can be attributed to several factors, such as increased musculoskeletal and neuromuscular impairments, pain, and fatigue, which are less prevalent or delayed in the healthy population [26] and sarcopenia [27].

The findings underscore the importance of lifelong monitoring, with a re-evaluation regarding the need for surgical interventions from early adulthood to adulthood in bCP patients with greater motor impairments.

CP subtype is a critical determinant of lifelong mobility: uCP patients generally maintain stable gait kinematics and walking speed into adulthood, while bCP patients have a higher frequency of clinically meaningful deterioration in walking speed, stride length, and passive range of motion.

The long-term findings of this study demonstrated that CP subtype is a critical determinant of lifelong mobility. Patients with uCP generally maintain stable gait kinematics and functional ambulation into adulthood, while bCP patients are more susceptible to a clinically meaningful decline in gait quality. Importantly, the observation of a universal, progressive decline in normalized walking speed across both subtypes highlights that even individuals with relatively preserved gait kinematics are not immune to functional mobility loss, underscoring the need for regular, lifelong monitoring of walking speed as a key clinical marker. These findings support a care model that emphasizes early identification and management of musculoskeletal deformities, individualized rehabilitation strategies, and proactive surveillance of all individuals with CP, with intensified monitoring and intervention for those with bilateral involvement to mitigate the risk of mobility decline and loss of independence.

The present study has several limitations that should be acknowledged. First, the retrospective design and the inclusion of only patients who repeated CGA in adulthood could have introduced a selection bias. While the sensitivity analysis indicated that participants with adulthood data had a baseline gait severity comparable to those lost to follow-up, the longitudinal trajectories observed in adulthood may reflect the experience of a clinically engaged cohort and should be generalized with caution. This study was not powered to perform stratified analyses by baseline motor function severity (GMFCS level II-III: n = 17 baseline, n = 5 adulthood); therefore, the potential influence of this factor on the observed outcomes could not be determined. This cohort likely over-represents ambulatory patients who stay engaged with healthcare services, and the majority of the patients were functional (GMFCS I), even in the bCP group (56%). Consequently, the functional status of patients lost to follow-up is unknown; we cannot ascertain how many experienced a loss of walking ability or maintained stable function without further clinical need, and thus these findings cannot be generalized to those with more severe impairments (GMFCS III-IV). Second, the study spans a 31-year period, during which the standard of care for orthopedic and rehabilitation management of CP evolved. Thus, while treatments were patient-specific and aligned with the clinical practice of their eras, the included cohort was heterogenous in terms of orthopedic interventions, and the notably higher number of surgeries in the bCP group compared to the uCP group represents a significant confounding variable when comparing long-term functional outcomes. This heterogeneity precludes a detailed analysis of the long-term comparative effectiveness of different treatment approaches and complicates the attribution of functional decline to natural history versus the cumulative burden of surgical intervention. Additionally, the potential role of each treatment type as a confounder could not be assessed, as the non-surgical treatments were not systematically collected. Third, the focus on CGA limits the clinical interpretation, as critical outcomes such as muscle strength, pain, or patient-reported outcomes were not assessed. Most critically, the absence of patient-reported outcome measures (PROMs) means we could not assess patient-perceived changes in function, pain, or quality of life, which are fundamental to understanding the real-world impact of the observed gait changes.

Future research should aim to establish larger, more comprehensive multicentric cohorts that intentionally include a greater representation of individuals with more severe impairments (GMFCS levels III-IV) to fully capture the spectrum of lifelong mobility. Furthermore, expanding the scope of assessment to include PROMs is essential to evaluating the correlation between objective gait changes and patients’ own experiences of function, mobility, and quality of life. Finally, future investigations should look beyond clinical factors to explore social and environmental determinants—such as access to services, physical activity opportunities, and psychosocial support—that underpin the successful maintenance of mobility in adulthood.

## 5. Conclusions

Individuals with spastic CP achieve significant improvements in gait quality by early adulthood but face a clinical decline during adulthood, with those having bCP being nearly five times more likely to experience functional deterioration. Given the limited adult sample size, this quantitative risk estimate should be interpreted with caution. These findings highlight the critical need for specialized adult CP care that specifically addresses the vulnerable transition from early adulthood to later adulthood. In this context, the need for surgical interventions in this period in individuals with greater motor impairments (GMFCS II–III) should be considered, taking into account their personal and professional engagements (e.g., employment and family responsibilities).

## Figures and Tables

**Table 1:** Demographics and clinical characteristics on the first and last visit.

	bCP (n = 39)		uCP (n = 44)		Group Comparison		
	1st Visit	Last Visit	1st Visit	Last Visit	*p*-Value	95%CI	ES
**Demographics**							
Female, n (%)	21 (48%)		13 (33%)		0.268	−9 to 38%	0.092
Age, years	9.8 (3.3)	23.6 (5.1) ***	10.2 (3.0)	24.0 (5.8) ***	0.602	−1.7 to 1.0	0.067
Height, m	1.31 (0.16)	1.64 (0.88) ***	1.40 (0.18)	1.71 (0.10) ***	0.029	−0.16 to −0.09	0.764
Weight, kg	32.5 (14.7)	61.5 (13.5) ***	34.7 (13.4)	68.3 (16.4) ***	0.478	−8.4 to 4.0	0.454
BMI, kg/m^2^	18.0 (4.8)	23.0 (4.9) ***	17.2 (3.3)	23.3 (4.6) ***	0.385	−1.0 to 2.6	0.780
**GMFCS levels**							
I, n (%)	22 (56%)	20 (51%) ^ns^	44 (100%)	44 (100%) ^ns^	<0.001	−62 to −27%	1.622
II, n (%)	10 (27%)	9 (23%) ^ns^	0 (0%)	0 (0%) ^ns^	0.001	10 to 42%	0.793
III, n (%)	7 (17%)	10 (26%) ^ns^	0 (0%)	0 (0%) ^ns^	0.011	3 to 32%	0.487
**Follow-up**							
Time, years	13.9 (6.0)		13.8 (5.3)		0.946	−2.4 to 2.6	0.015
Nb CGA, n	3.1 (0.7)		3.0 (0.6)		0.501	−0.2 to 0.4	0.150
**Surgical events**							
No surgery, n (%)	7 (18%)		10 (23%)		0.713	−23 to 13%	0.713
1 surgery, n (%)	12 (31%)		24 (54%)		0.051	−47 to −1%	0.289
>1 surgery, n (%)	20 (51%)		10 (23%)		0.013	6 to 51%	0.461
Multilevel surgery, n (%)	17 (44%)		12 (27%)		0.105	−6 to 39%	0.132
Osteotomy femur, n (%)	14 (36%)		9 (20%)		0.189	−6 to 37%	0.134
Osteotomy tibia, n (%)	9 (23%)		11 (25%)		>0.999	−22 to 18%	<0.001
Patella lowering, n (%)	10 (26%)		1 (2%)		0.005	−40 to −7%	0.595
T/M Lengthening, n (%)	14 (36%)		11 (25%)		0.401	−11 to 33%	0.053
T/M Transfer, n (%)	29 (74%)		28 (64%)		0.416	−11 to 32%	0.050
BTX, n (%)	20 (51%)		20 (45%)		0.756	−18 to 30%	0.007
**Gait outcomes**							
mGPS, deg	11.3 (4.7)	9.8 (4.4) *	8.7 (2.4)	7.1 (1.3) ***	0.003	0.9 to 4.3	0.856
Deteriorated, n (%)	5 (13%)		2 (5%)		0.338	−6 to 23%	0.069
NWS, s^−1^	1.3 (0.5)	1.0 (0.4) **	1.5 (0.3)	1.3 (0.2) ***	0.028	−0.4 to −0.0	0.814
Deteriorated, n (%)	23 (52%)		24 (62%)		0.530	−33 to 14%	0.030

T is tendon, M is muscle, and BTX is botulinum toxin. For the longitudinal comparison of gait parameters from the 1st and the last CGA, a paired Student *t*-test was performed in each group (uCP and bCP), and results were reported as follows: ***: *p* < 0.001, **: *p* < 0.01; *: *p* < 0.05; ns: *p* ≥ 0.05. Unpaired Student *t*-tests were performed to compare characteristics between the uCP and bCP groups, reported with 95% confidence intervals (95%CI) and effect sizes (ES). For the demographics and clinical characteristics group comparison, the 1st visit values were compared, and for the surgical events, the value recorded during the last visit was used. Gait quality was evaluated with the modified Gait Profile Score (mGPS), and gait function was evaluated with normalized walking speed (NWS). To identify deteriorated cases, a minimal clinically important difference (MCID) of 1.6° for the mGPS [19] and 0.20 s^−1^ for the NWS [20] was applied.

**Table 2:** Longitudinal gait outcomes from childhood to adulthood in unilateral and bilateral cerebral palsy.

Variable	Life Stage	bCP (n = 39)	uCP (n = 44)	*p*-Value	95%CI	ES
**Age, years**	Childhood	9.1 [8.3; 10.9] ^a^	9.1 [7.1; 11.2] ^a^	0.667	−1.0 to 1.5	0.050
	Adolescence	15.0 [13.9; 15.8] ^b^	14.9 [14.4; 15.5] ^b^	0.882	−0.8 to 0.6	0.151
	Early Adulthood	20.0 [18.9; 22.0] ^c^	20.1 [19.0; 22.1] ^c^	0.970	−0.9 to 0.9	0.209
	Adulthood	27.3 [25.9; 30.9] ^d^	28.6 [25.4; 31.7] ^d^	0.956	−3.4 to 3.6	0.298
**Gait quality (mGPS), degrees**	Childhood	8.0 [7.1; 9.5] ^a^	11.3 [8.1; 15.3] ^a^	0.003	−4.9 to −0.9	0.319
	Adolescence	7.3 [6.1; 9.0] ^b^	8.8 [6.7; 11.8] ^b^	0.029	−2.9 to −0.2	0.241
	Early Adulthood	7.1 [6.0; 8.1] ^c^	8.6 [7.0; 10.8] ^c^	0.002	−2.9 to −0.7	0.326
	Adulthood	7.7 [6.4; 8.3] ^d^	7.6 [5.8; 10.1] ^d^	0.927	−2.3 to 1.2	0.254
**Gait function (NWS), s^−1^**	Childhood	1.6 [1.4; 1.8] ^a^	1.5 [1.2; 1.8] ^a^	0.010	0.1 to 0.4	0.150
	Adolescence	1.3 [1.2; 1.4] ^b^	1.2 [1.0; 1.4] ^b^	0.184	−0.1 to 0.2	0.114
	Early Adulthood	1.3 [1.2; 1.4] ^c^	1.2 [1.0; 1.3] ^c^	0.047	0.0 to 0.2	0.186
	Adulthood	1.2 [1.1; 1.4] ^d^	1.1 [0.9; 1.2] ^d^	0.927	0.0 to 0.4	0.309

Paired Wilcoxon tests were used to compare data across life stages—defined as childhood (female: <13.5 years; male: <15.5 years), adolescence (female: 13.5–18 years; male: 15.5–18 years), early adulthood (18–25 years), and adulthood (>25 years)—for each patient. Gait quality was evaluated with the modified Gait Profile Score (mGPS), and gait function was evaluated with normalized walking speed (NWS). In these longitudinal analyses, a participant contributed data only for the specific stages in which they were assessed; missing a stage resulted in exclusion from that specific stage’s comparison but not from others. This led to different sample sizes for each life stage, reported as ^a^ n = 38 uCP, n = 35 bCP; ^b^ n = 31 uCP, n = 31 bCP; ^c^ n = 42 uCP, n = 39 bCP; and ^d^ n = 17 uCP, n = 14 bCP.

## Data Availability

The data supporting the findings of this study are not openly available due to sensitivity concerns. They are accessible from the corresponding author upon reasonable request. The data are stored in a controlled-access repository at Geneva University Hospitals, Switzerland.

## Abbreviations

The following abbreviations are used in this manuscript:

CP	Cerebral palsy
GMFCS	Gross Motor Function Classification System
uCP	Unilateral cerebral palsy
bCP	Bilateral cerebral palsy
mGPS	Modified Gait Profile Score
NWS	Normalized walking speed
MCID	Minimal clinically important difference
